# Soil microbial communities response to different fertilization regimes in young *Catalpa bungei* plantation

**DOI:** 10.3389/fmicb.2022.948875

**Published:** 2022-08-08

**Authors:** Zhuizhui Guan, Daiyi Lin, Dong Chen, Yundan Guo, Yizeng Lu, Qingjun Han, Ningning Li, Yan Su, Jiyue Li, Junhui Wang, Wenjun Ma, Quan Qiu, Qian He

**Affiliations:** ^1^Guangdong Key Laboratory for Innovative Development and Utilization of Forest Plant Germplasm, South China Agricultural University, Guangzhou, China; ^2^Shandong Provincial Center of Forest and Grass Germplasm Resources, Jinan, China; ^3^Research Institute of Forestry, Chinese Academy of Forestry, Beijing, China

**Keywords:** *Catalpa bungei*, integration of water and fertilizer, microbial community, diversity and composition, functional taxa

## Abstract

Fertilization is a fundamental aspect of global forest management that enhances forest productivity and drastically affects soil microbial communities. However, few studies have investigated the differences and similarities in the responses of below-ground microbial communities to different fertilization schemes. The effects of fertilization regimes on the composition and diversity of soil fungal and bacterial communities were investigated in a young *Catalpa bungei* plantation in Shandong Province, Eastern China. Soil microbial communities were assessed undergoing three types of fertilization: (i) no fertilization (CK), (ii) hole fertilization (HF), and (iii) the integration of water and fertilizer (WF). We further analyzed the effects of soil depth (i.e., 0–20 and 20–40 cm) on the structure of soil microbial communities. Our results indicated that the diversity of bacteria (e.g., Chao1 and Shannon indices) reduced undergoing fertilization, and WF had a higher negative impact on bacterial diversity than HF. A lower bacterial diversity was observed in the subsoil compared to the topsoil. In contrast to bacterial diversity, fungal diversity had a slightly increasing trend in the fertilized environments. The primary bacterial function was metabolism, which was independent of fertilization or soil depth. Among fungal functional guilds, symbiotic soil fungi decreased obviously in the fertilized stand, whereas saprotrophic fungi increased slowly. According to the structural equation models (SEM), the diversity and composition of bacterial and fungal communities were jointly regulated by soil nutrients (including N and P contents) directly affected by fertilization and soil layer. These findings could be used to develop management practices in temperate forests and help sustain soil microbial diversity to maintain long-term ecosystem function and services.

## Introduction

It is generally believed that there is an interaction among the plants, soil, and microorganisms, such as the habitat and nutrients required for plant and microbe growth are supplied by the soil, while most of the nutrients in the soil are sponsored by plant growth and microbial activities (Patel et al., 2015, 2021). As a prominent member of the soil ecosystem, the soil microbiome plays a pivotal role in maintaining the material cycle and nutrient flow in the soil function. Soil microbial functional diversity is also an indispensable component in the soil health and quality evaluation system, including processes such as organic matter decomposition, humus synthesis, and nutrient transformation and recycling (Fan et al., 2013).

Fertilization has consistently been shown to be a highly effective method for enhancing tree productivity (Shi et al., 2017). Soil microbial communities exhibit a wide range of compositions depending on the vegetation, soil type, management strategy, and fertilization regime (Zhang et al., 2014; Kivlin and Hawkes, 2016; Nie et al., 2018). Most of the previous studies are focused on the response of the soil microbial community to regular fertilization (e.g., N or P addition). For example, Ling et al. (2017) found that both N and P additions altered the bacterial community structure in a semi-arid steppe. He et al. (2013) highlighted that the higher N fertilization was not suitable for the growth and improvement of functional diversity of the soil microbial community. Hamel et al. (2010) suggested that long-term P input affected soil fungal and bacterial diversity but not the fungal community of arbuscular mycorrhiza (AM) in Alfalfa. However, these results only explored the effects of N and P additions on microbial populations, and how soil microbes ultimately respond to N, P, and K coupled fertilization remained poorly understood. Furthermore, the changes in microbial abundance and community composition with soil properties are bound to be affected by the vertical distribution of soil profiles (Lin et al., 2022). However, most studies focus on the effect of fertilization on the microbial community of the surface soil (e.g., 0–20 cm; Griffiths et al., 2012; Fierer et al., 2012a; Zhou et al., 2015), and the responses of the longitudinal variation of soil and microbial properties to soil depth undergoing fertilization are relatively rare reported.

It is widely recognized that the integration of water and fertilizer has significant effects on promoting the growth of seedlings (Zhang et al., 2019), increasing crop yield (Hu et al., 2010; Chen et al., 2018), and improving fruit quality (Liu et al., 2020) compared with conventional fertilization. Most reports only focus on the effects of the integration of water and fertilizer on the agricultural ecosystem (Kumar et al., 2021; Lu et al., 2021; Zhao et al., 2021), while there are a few cases of applying this technology to forest trees (He et al., 2021). Currently, one study found that when fertilizing *Poplar tomentosa*, the utilization of water and fertilizer integration might be able to reduce the amount of fertilizer used compared with the conventional fertilization scheme (Xi et al., 2017). The integration of water and fertilizer has also been reported to alter soil nutrients and enzyme activities (Li et al., 2022a). However, it is not clear how the application of water and fertilizer integration affects the structure of the soil microbial community. In addition, understanding the effects of different fertilization regimes on the function of soil microbiome can help predict soil functioning and maintain ecosystem sustainability.

In this study, we explored the shifts in abiotic and biotic factors between regular fertilization (e.g., hole fertilization) and the integration of water and fertilizer in young *C. bungei* plantations in Eastern China. First, we determined the soil’s physical and chemical parameters in two soil layers under each fertilization regime. Next, we tracked the compositional and functional changes in the microbial communities across fertilization regimes. Last, we identified links between soil properties and microbial community shifts. This study intends to explore the following questions: (1) how bacterial and fungal community composition, diversity, and function respond to different fertilization regimes; (2) explore the key soil drivers that cause soil bacterial and fungal community and composition along the soil vertical profile undergoing fertilization. Investigating the response mechanisms of the composition and functional groups of the soil microbiota to different fertilization schemes will be helpful for evaluating and improving the soil quality in the *C. bungei* plantation, thereby providing scientific guidelines for the fertilization of trees.

## Materials and methods

### Study site and tree species

**Figure 1:**
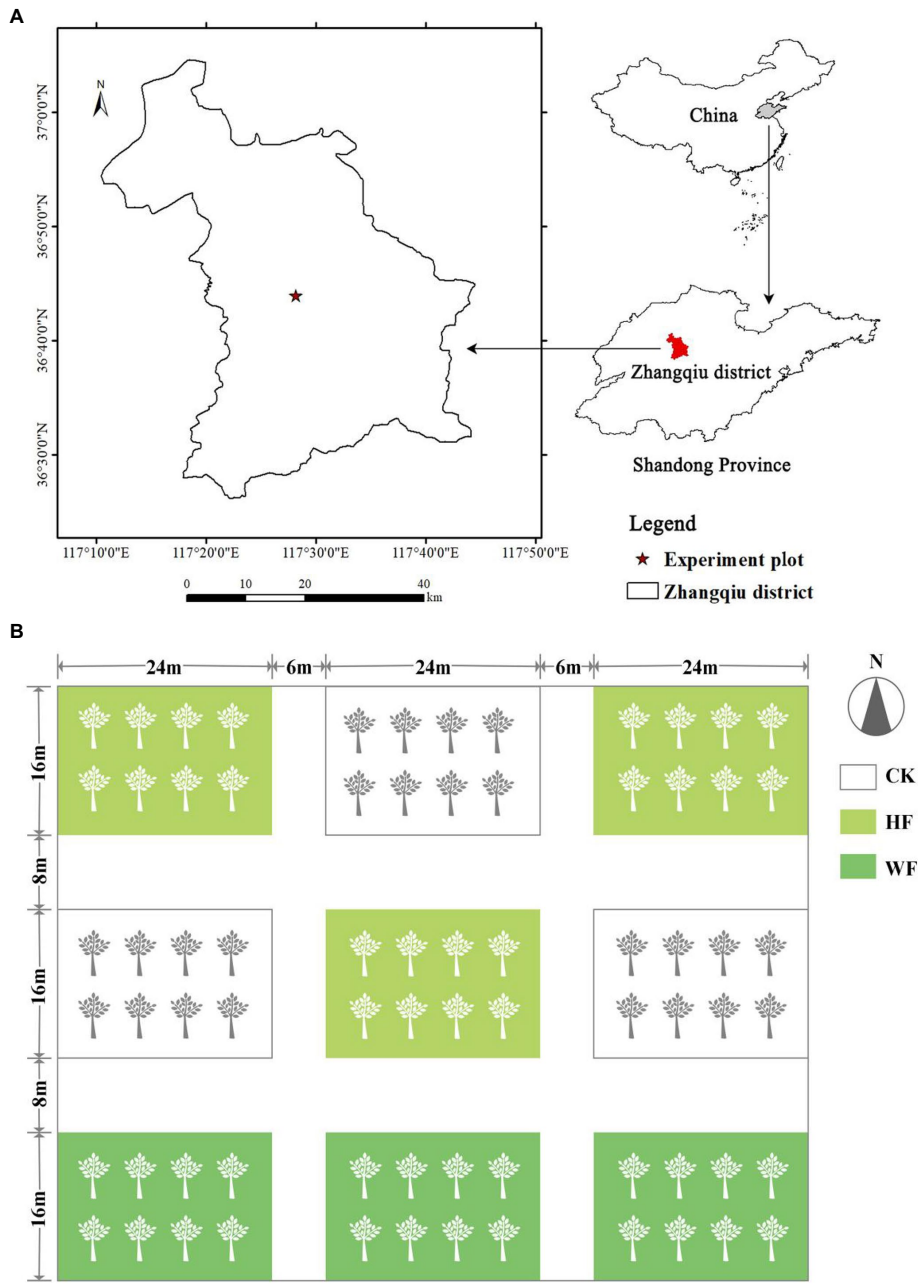
Study area **(A)** and sampling sites **(B)** in the Zhangqiu district, Shandong Province of China. White in the box indicates no fertilization (CK), light green indicates hole fertilization (HF), and dark green indicates integration of water and fertilizer (WF).

The experiment was conducted at the Jujube Preservation Warehouse in Zhangqiu District (36°25′–37°09′ N, 117°10′–117°35′ E), Jinan City, Shandong Province, China (Figure 1A). The site had a temperate monsoon climate, with a mean annual temperature of 12.8°C, the highest mean monthly temperature of 27.2°C (July), and the lowest mean monthly temperature of-3.2°C (January). The frost-free period is of 192 days, the mean annual precipitation is 600.8 mm, and the annual sunshine duration is 2647.6 h. The soil types are mainly cinnamon and fluvo-aquic soils (Zhao et al., 2020).

Pure plantations of *C. bungei* were selected, and established in March 2017 with 2-year-old clones (“9–1”) in a 3.0 × 4.0 m planting grid. In total, 0.8 ha of *C. bungei* were established in 2017. No tending had been applied since planting. Three months after afforestation, the mean tree height and diameters at breast height (DBH) were 4.2 m and 4.0 cm, respectively. A growing season spans from May to September and lasts for 130 to 150 days.

### Fertilization

In early May 2018 (before bud break), nine plots (approximately 400 m^2^/ plot) were randomly chosen and fertilized on the plantation. We designed two fertilization schemes: integration of water and fertilizer (WF) and hole fertilization (HF), and no fertilization (CK) was a control treatment. Each type of fertilization was randomly distributed in the plots with three replicates, and isolation zones were set between the plots (Figure 1B). The first fertilizer was applied in May 2018, and the total amount of fertilizer was N (24 g/ tree), P_2_O_5_ (8 g/ tree), and K_2_O (16 g/ tree), followed by an annual increase of 20% based on our past experience for fertilization. All fertilizer purchase channels in the experiment were from the merchants on the Taobao website. The name of the merchant was Xuanming Horticulture. HF was applied once in May. We dug two holes vertically at 50 cm to the south and north of the tree. The hole had a diameter of 20 cm and a depth of 30 cm. Then, we put an equal amount of fertilizer into the hole. The total amount of WF fertilizer was divided into 12 times and applied once every 10 days, starting on May 1st and ending on September 1st *via* intelligent drip irrigation equipment (HN-BXE, Huinong Automation Company, China). We measured the growth of trees under different fertilization conditions in October 2021 (Supplementary Table S1). Air temperature and precipitation data recorded by an on-site meteorological station for the period 2017–2021 are displayed in Supplementary Figure S1.

### Soil sampling and measurements

Soil samples were collected in October 2021, and five 0–20 cm (surface layer) and 20–40 cm (subsurface layer) samples were randomly collected along the diagonal of each plot. Samples of each soil layer were sieved through a 2 mm mesh to remove litter, roots, and other visible items, and mixed evenly. The composite soil samples were transported to the laboratory in a cooler at −4°C, then one portion was immediately frozen at −80°C for subsequent microbiological analysis. The remaining soil was divided into two equal parts: one was transferred to the refrigerator (−4°C) for enzyme activity analysis, and the other was air-dried for physicochemical analysis. In addition, three soil cores (diameter, 5.5 cm) were randomly collected from each plot and combined into composite samples for the respective plots. The soil cores were used to measure soil moisture content (SMC). We also determined the soil pH, soil organic carbon (SOC), soil organic matter (SOM), total nitrogen (TN), total phosphorous (TP), total potassium (TK), available nitrogen (AN), available phosphorous (AP), and available potassium (AK) using standard procedures (Lu, 2000). Soil pH was measured using the potentiometric method (1,2.5, soil:water ratio; Lu, 2000). SOM was measured using the potassium dichromate volumetric method (Lu, 2000). SOC equaled SOM divided by 1.724. TN was measured using the acid digestion-indophenol blue colorimetric method (Lu, 2000). TP was measured using the acid digestion-molybdenum antimony resistance colorimetric method (Lu, 2000). TK and AK were measured using the acid digestion-flame atomic absorption method (Lu, 2000). AN was measured by the potassium chloride leaching-indophenol blue colorimetric method (Lu, 2000). AP was measured by hydrochloric acid, ammonium fluoride leaching/sodium bicarbonate leaching-molybdenum antimony anti-colorimetric method (Lu, 2000). The C:N ratio was calculated as the ratio between the SOC and TN.

### Soil enzyme activity and microbial biomass CNP

Catalase activity was measured by adding 5 ml of 0.3% hydrogen peroxide solution to a 2 g soil sample, shaking for 20 min (200 r/min), then centrifuging, filtering, and taking 3–4 ml solution for colorimetric analysis (Lu, 2000). To measure acid phosphatase activity, we added 4 ml of buffer and 1 ml of sodium p-nitrophenyl phosphate solution (100 mol/l) to 1 g of fresh soil, followed by incubation at constant temperature for 30 min (30°C), and then added 1 ml of CaCl_2_ (2 mol/l) and 4 ml of NaOH (0.2 mol/l) until the reaction stopped, and finally filtered and colorimetric (Lu, 2000). Urease activity was assayed in 10% urea and citric acid buffer (pH 6.7, 90 min at 37°C), and indophenol was used to measure the NH_4_^+^ released (Lu, 2000). To measure the sucrase activity, we added 15 ml of sucrose solution (8%), 5 ml of phosphate buffer (pH 5.5), and 0.25 ml of toluene to 2 g of fresh soil, respectively, followed by constant temperature incubation (37°C, 24 h), and then added 3 ml of 3,5-dinitrosalicylic acid, followed by boiling water bath for 5 min, cooling, and finally, colorimetry is performed (Lu, 2000). We referred to the methods provided by Lu (2000) for the determination of microbial CNP. Microbial biomass C and N were determined using potassium sulfate extraction, the fumigation–TOC method. Microbial biomass P was determined using ammonium fluoride hydrochloric acid/sodium bicarbonate extraction, fumigation-molybdenum-antimony anti-colorimetric method. Microbial biomass C:N, C:P, and N:P ratios represented the ratio between microbial biomass CNP.

### Molecular analysis of soil microorganisms

Total genomic DNA from samples was extracted using the CTAB method. DNA concentration and purity were monitored on 1% agarose gel. According to the concentration, DNA was diluted to 1 ng/μl using sterile water. The V3–V4 region of the bacterial 16S rRNA gene was amplified using the primers 341F (5′–CCTAYGGGRBGCASCAG–3′) and 806R (5′–GGACTACNN GGGTATCTAAT–3′; Wang et al., 2019). The fungal internal transcribed spacer (ITS) was amplified using the primers ITS1-1F-F (5′–CTTGGTCATTTAGAGGAAGTAA–3′) and ITS1-1F-R (5′–GCTGCGTTCTTCATCGATGC–3′; Bokulich et al., 2018). All PCR reactions were carried out with 15 μl of Phusion^®^ High-Fidelity PCR Master Mix (T100PCR, Bio-Rad, United States); 1 μl of forward primers (2 μM/μl) and reverse primers (2 μM/μl), respectively; and about 10 ng template DNA. Thermal cycling consisted of initial denaturation at 98°C for 1 min, followed by 30 cycles of denaturation at 98°C for 10 s, annealing at 50°C for 30 s, and finally elongation at 72°C for 30 s and 72°C for 5 min. PCR products were separated on a 2% agarose/1X TAE gel and purified with a Qiagen Gel Extraction Kit (Qiagen, Germany).

Sequencing libraries were generated using the TruSeq^®^ DNA PCR-Free Sample Preparation Kit (Illumina, United States) following the manufacturer’s recommendations, and index codes were added. The library quality was assessed on the Qubit@ 2.0 Fluorometer (Life Technologies, CA, United States). At last, the library was sequenced on an Illumina NovaSeq platform (Novaseq 6,000, Illumina, San Diego, CA, United States) and 250 bp paired-end reads were generated. The number of sequences reached 50,000.

The analysis was conducted following the “Atacama soil microbiome tutorial” of Qiime2docs and the customized program scripts.^1^ Briefly, raw data FASTQ files were imported into the format that could be operated by the QIIME2 system using the qiime tools import program. Demultiplexed sequences from each sample were quality filtered and trimmed, de-noised, merged, and then the chimeric sequences were identified and removed using the QIIME2 dada2 plugin to obtain the feature table of amplicon sequence variant (ASV; Callahan et al., 2016). The QIIME2 feature-classifier plugin was then used to align ASV sequences to a pre-trained GREENGENES 13_8 99% database (trimmed to the V3V4 region bound by the 341F/806R primer pair or ITS region bound by the ITS1-1F-F/ITS1-1F-R primer pair) to generate the taxonomy table (Bokulich et al., 2018). The default UNITE database version of ITS was 8.2. Any contaminating mitochondrial and chloroplast sequences were filtered using the QIIME2 feature-table plugin.

### Network analysis

The relevant network was mainly drawn using the “*igraph*” package in R. Based on the relative abundance between species, the interaction between species was obtained by performing the Spearman analysis. Then, the interaction network of species was constructed using a visualization software. The analysis looked for mutually antagonistic or synergistic species, thereby collecting information on mutual cooperation or inhibition of microbial populations.

### Data analysis

Differences in several factors, including soil physicochemical properties, soil enzyme activities, and soil microbial biomass CNP, among fertilization types, were determined using a two-way analysis of variance (ANOVA) and the Kruskal-Wallis test. The above statistical analyses were performed using SPSS 25.0 (IBM Company, United States).

Diversity metrics were calculated using the core-diversity plugin within QIIME2. Feature-level alpha diversity indices, such as observed OTUs, Chao1, Shannon, and Simpson indices, were calculated to estimate the microbial diversity within an individual sample. Beta diversity distance measurements, such as Bray-Curtis, were performed to investigate the structural variation of microbial communities across samples and then visualized *via* nonmetric multidimensional scaling (NMDS; Yoshiki et al., 2013). Redundancy analysis (RDA) was performed to reveal the association of microbial communities in relation to environmental factors based on relative abundances of microbial species at different phyla levels using the R package “*vegan*” (Bokulich et al., 2018). LEfSe multilevel species difference discriminant analysis evaluated the groups that had a significant impact on sample differences, and LDA linear discriminant analysis screened for groups that had a significant effect on differences between groups (LDA score > 2). In addition, the potential KEGG Ortholog (KO) functional profiles of bacterial communities was predicted with PICRUSt2 (Langille et al., 2013). Functional prediction of bacteria was performed on the Tutools platform,^2^ a free online data analysis website. To determine whether the fungal functional guilds were affected by changes in fertilization types, the FUNGuild tool was used to classify each OTU into an ecological guild (Nguyen et al., 2016; Li et al., 2018; Gan et al., 2021). The number of OTUs indicated the richness of a guild, and the relative abundance of each guild was calculated by dividing the number of sequences of a specific guild by the total number of sequences (Li et al., 2018).

The effects of soil properties (including soil physicochemical properties, enzyme activities, and microbial biomass CNP) on the composition and diversity of soil fungal and bacterial communities were determined using a structural equation model (SEM) *via* the “*lavaan*” package in R, as reported in previous studies (Wardle et al., 2004; Li et al., 2018). SEM was used to evaluate the direct and indirect relationships among several factors, including soil physicochemical properties, soil enzyme activities, and microbial biomass CNP, and the composition and diversity of fungal and bacterial communities present in the soil, while simultaneously accounting for multiple drivers. Model fitness was measured using the chi-squared test (*p* > 0.05), root mean square error of approximation (RMSEA < 0.05), comparative fit index (CFI > 0.95), and goodness-of-fit index (GFI > 0.95). The path coefficient analysis was used to estimate the effects of all explanatory variables on the composition and diversity of soil microbial communities.

## Results

### Soil parameters in response to fertilization

For soil physicochemical properties, fertilization and soil layer had significant effects on SOM, SOC, and AN, but had no effects on SMC, pH, TK, and AK (Table 1). Fertilization increased SOM, SOC, and AN, but deeper soil decreased SOM, SOC, TN, TP, AN, and AP than the topsoil. For soil enzyme activity, fertilization significantly reduced the urease activity. The activities of acid phosphatase, urease, and sucrase in the topsoil were higher than in the subsoil. For microbial biomass, fertilization significantly reduced MBC in the topsoil but increased MBC in the subsoil. The MBC, MBN, and MBP gradually decreased with the deepening of the soil layer, but the ratio of microbial biomass increased gradually with the deepening of the soil layer.

**Table 1:** Soil physicochemical properties, soil enzyme activities, and microbial biomass CNP after fertilization treatments, measured in October 2021.

Soil properties	CK		HF		WF		F	S	F × S
	S20	S40	S20	S40	S20	S40			
SMC (%)	17.9 (1.6)a	17.8 (0.6)a	17.4 (0.3)a	17.8 (0.2)a	16.6 (1.5)a	17.4 (1.3)a	ns	ns	ns
pH (H_2_O)	7.49 (0.39)a	7.80 (0.29)a	7.47 (0.07)a	7.72 (0.10)a	7.54 (0.10)a	7.77 (0.13)a	ns	ns	ns
SOM (g·kg^−1^)	17.89 (1.85)ab	10.37 (0.93)c	18.17 (0.27)ab	13.58 (1.37)b	18.87 (1.15)a	18.36 (1.46)ab	^***^	^***^	^**^
SOC (g·kg^−1^)	10.38 (1.08)ab	6.02 (0.54)c	10.54 (0.16)ab	7.88 (0.80)bc	10.95 (0.67)a	10.65 (0.85)ab	^***^	^***^	^**^
TN (g·kg^−1^)	0.86 (0.05)a	0.62 (0.11)b	0.88 (0.02)a	0.71 (0.05)b	0.86 (0.07)a	0.73 (0.03)ab	ns	^***^	ns
TP (g·kg^−1^)	0.39 (0.02)a	0.23 (0.04)c	0.34 (0.06)ab	0.26 (0.01)bc	0.33 (0.02)ab	0.27 (0.02)abc	ns	^***^	^*^
TK (g·kg^−1^)	19.53 (0.18)a	19.25 (0.45)a	19.20 (1.54)a	19.00 (1.52)a	19.20 (0.77)a	19.57 (0.40)a	ns	ns	ns
AN (mg·kg^−1^)	48.26 (3.82)ab	33.25 (7.33)c	55.66 (1.44)a	38.80 (5.41)bc	54.27 (3.20)ab	45.49 (4.92)b	^*^	^***^	ns
AP (mg·kg^−1^)	7.53 (2.77)a	1.28 (0.74)b	7.21 (3.87)a	1.39 (0.84)b	6.24 (2.74)a	0.65 (0.14)b	ns	^**^	ns
AK (mg·kg^−1^)	157.92 (11.96)a	104.09 (7.11)a	163.73 (35.42)a	148.72 (31.89)a	143.26 (33.67)a	122.71 (15.74)a	ns	ns	ns
C:N ratio	12.13 (1.40)ab	9.74 (1.28)b	12.02 (0.42)b	11.23 (1.72)b	12.70 (0.48)ab	14.52 (1.18)a	^*^	ns	^*^
Acid phosphatase (μg·g^−1^·h^−1^)	6.04 (0.10)a	5.24 (0.21)b	5.53 (0.39)ab	5.22 (0.46)b	5.44 (0.05)ab	5.17 (0.50)b	ns	^*^	ns
Urease (μg·g^−1^·h^−1^)	13.27 (1.70)a	9.76 (3.89)b	9.95 (0.19)b	8.23 (2.69)b	9.36 (0.52)b	6.47 (2.26)c	^*^	^*^	ns
Catalase (mg·g^−1^·20 min^−1^)	4.97 (0.28)a	5.21 (0.26)a	4.92 (0.05)a	5.17 (0.14)a	4.82 (0.29)a	5.28 (0.18)a	ns	ns	ns
Sucrase (mg·g^−1^·d^−1^)	6.06 (0.88)a	4.49 (0.58)b	6.73 (0.49)a	5.62 (0.82)b	6.41 (1.24)a	5.91 (0.77)ab	ns	^*^	ns
MBC (mg·kg^−1^)	106.65 (15.18)a	37.72 (9.37)c	69.48 (17.78)b	43.64 (15.02)c	51.61 (19.79)bc	47.19 (36.60)c	^*^	^**^	^*^
MBN (mg·kg^−1^)	19.62 (3.84)a	5.78 (0.75)b	13.19 (3.19)a	3.88 (1.56)b	12.82 (2.80)a	6.66 (1.82)b	ns	^***^	ns
MBP (mg·kg^−1^)	4.27 (1.67)a	0.75 (0.43)b	3.47 (1.85)a	0.59 (0.33)b	3.65 (2.11)a	0.31 (0.04)b	ns	^***^	ns
MBC:MBN ratio	5.48 (0.51)bc	6.75 (2.58)ab	5.37 (1.17)bc	11.41 (0.64)a	4.01 (1.31)c	6.32 (4.26)ab	ns	^**^	ns
MBC:MBP ratio	28.11 (13.50)c	68.07 (46.36)b	22.33 (7.49)c	123.85 (78.50)a	18.11 (8.41)c	161.02 (71.51)a	^*^	^*^	ns
MBN:MBP ratio	5.03 (1.97)c	10.28 (4.27)b	4.21 (1.17)c	11.33 (7.48)b	4.62 (1.89)c	22.16 (7.52)a	^*^	^*^	ns

SMC, soil moisture content; SOM, soil organic matter; SOC, soil organic carbon; TN, total nitrogen; TP, total phosphorus; TK, total potassium; AN, available nitrogen; AP, available phosphorus; AK, available potassium; C:N ratio, the ratio between the SOC and TN; MBC, microbial biomass carbon; MBN, microbial biomass nitrogen; MBP, microbial biomass phosphorus; F, fertilization, including CK, HF, and WF; CK, no fertilization; HF, hole fertilization; WF, integration of water and fertilizer; S, soil layer, including S20 and S40; S20, surface soil (0–20 cm); S40, subsurface soil (20–40 cm); F × S, interaction of fertilization and soil layer. Values are means with SE in parentheses (*n* = 3). Values followed by different letters (e.g., a, b and c) in the same row are significantly different among treatments with *P* < 0.05 (Kruskal-Wallis test). ns, no significant (*P* > 0.05).

* significance at the level of 0.05.

** significance at the level of 0.01.

*** significance at the level of 0.001, respectively.

### Composition of bacterial and fungal community in response to fertilization

**Figure 2:**
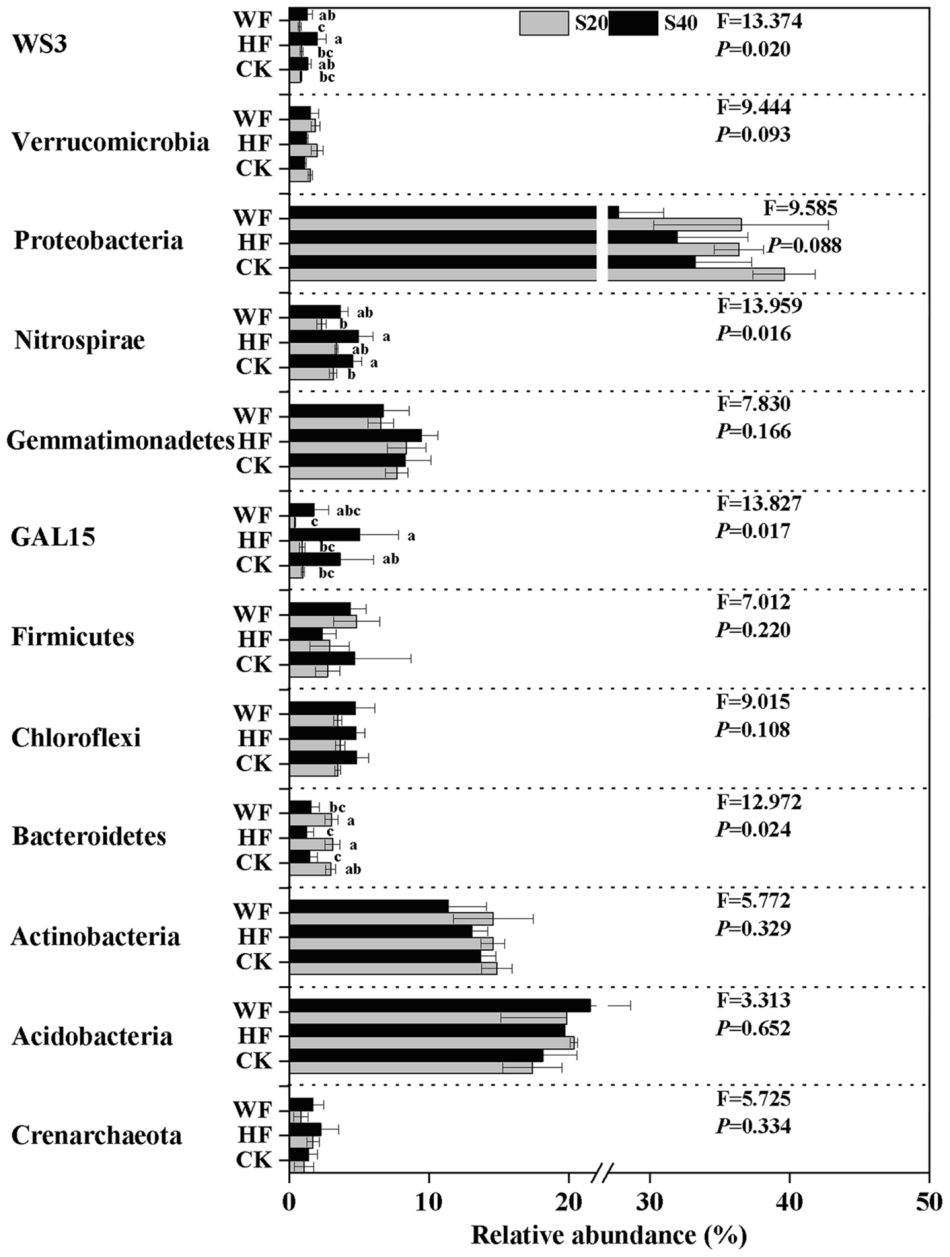
Relative abundance of bacteria group at phylum level under different fertilization treatments. The groups accounting for ≥1% are shown, while those at <1% and unclassified are not shown. F- and *p*-values of each phylum under different fertilization treatments are from the results of the Kruskal-Wallis test. Values followed by different letters are significantly different among treatments with *P* < 0.05 (*n* = 3). CK, no fertilization; HF, hole fertilization; WF, integration of water and fertilizer; S20, surface soil (0–20 cm); S40, subsurface soil (20–40 cm). The break is based on the median value of relative abundance.

**Figure 3:**
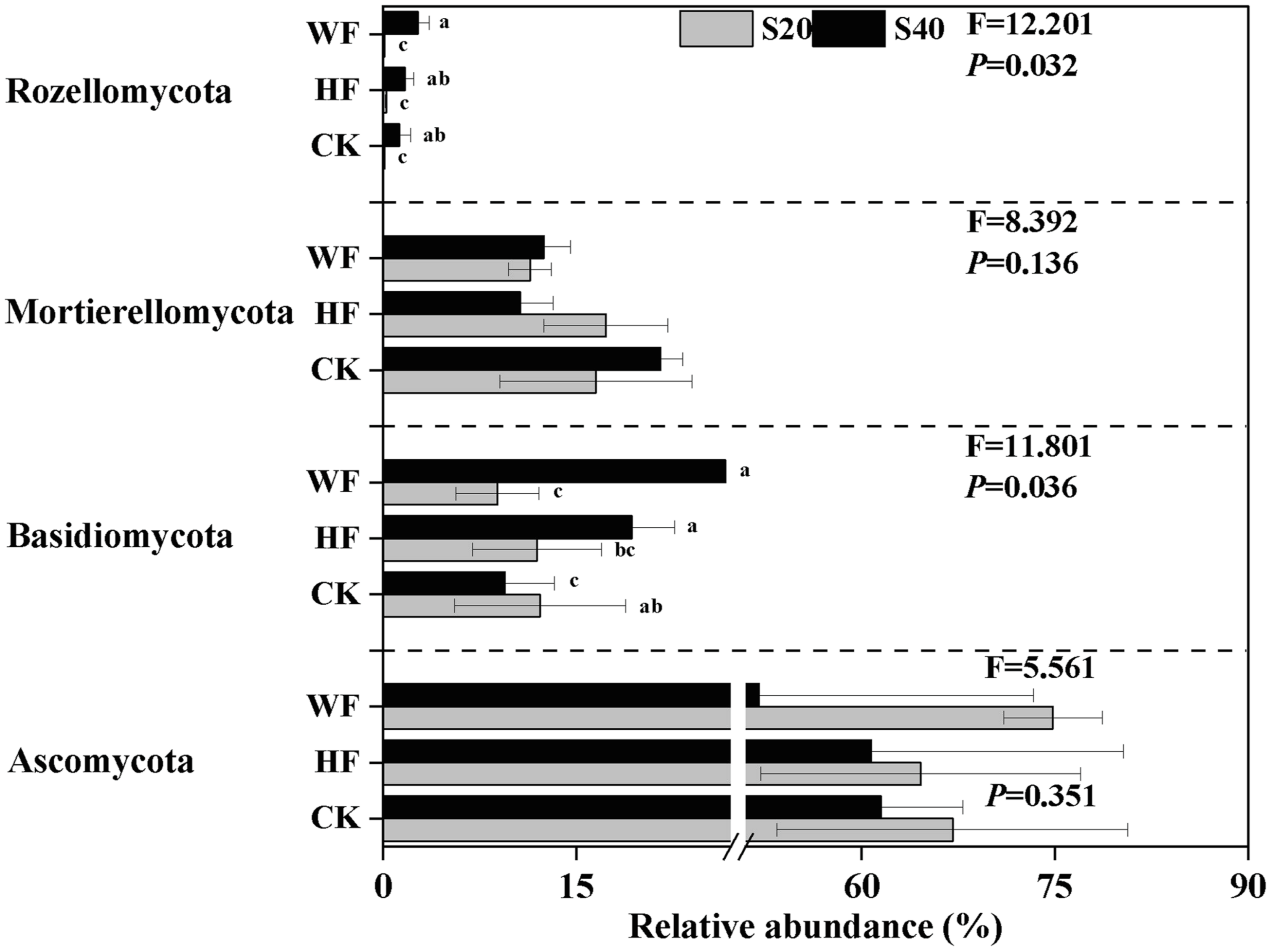
Relative abundance of fungi group at phylum-level under different fertilization treatments. The groups accounting for ≥1% are shown, while those at <1% and unclassified group are not shown. F- and *p*-values of each phylum under different fertilization treatments are from the results of the Kruskal-Wallis test. Values followed by different letters are significantly different among treatments with *P* < 0.05 (*n* = 3). CK, no fertilization; HF, hole fertilization; WF, integration of water and fertilizer; S20, surface soil (0–20 cm); S40, subsurface soil (20–40 cm). The break is based on the median value of relative abundance.

**Figure 4:**
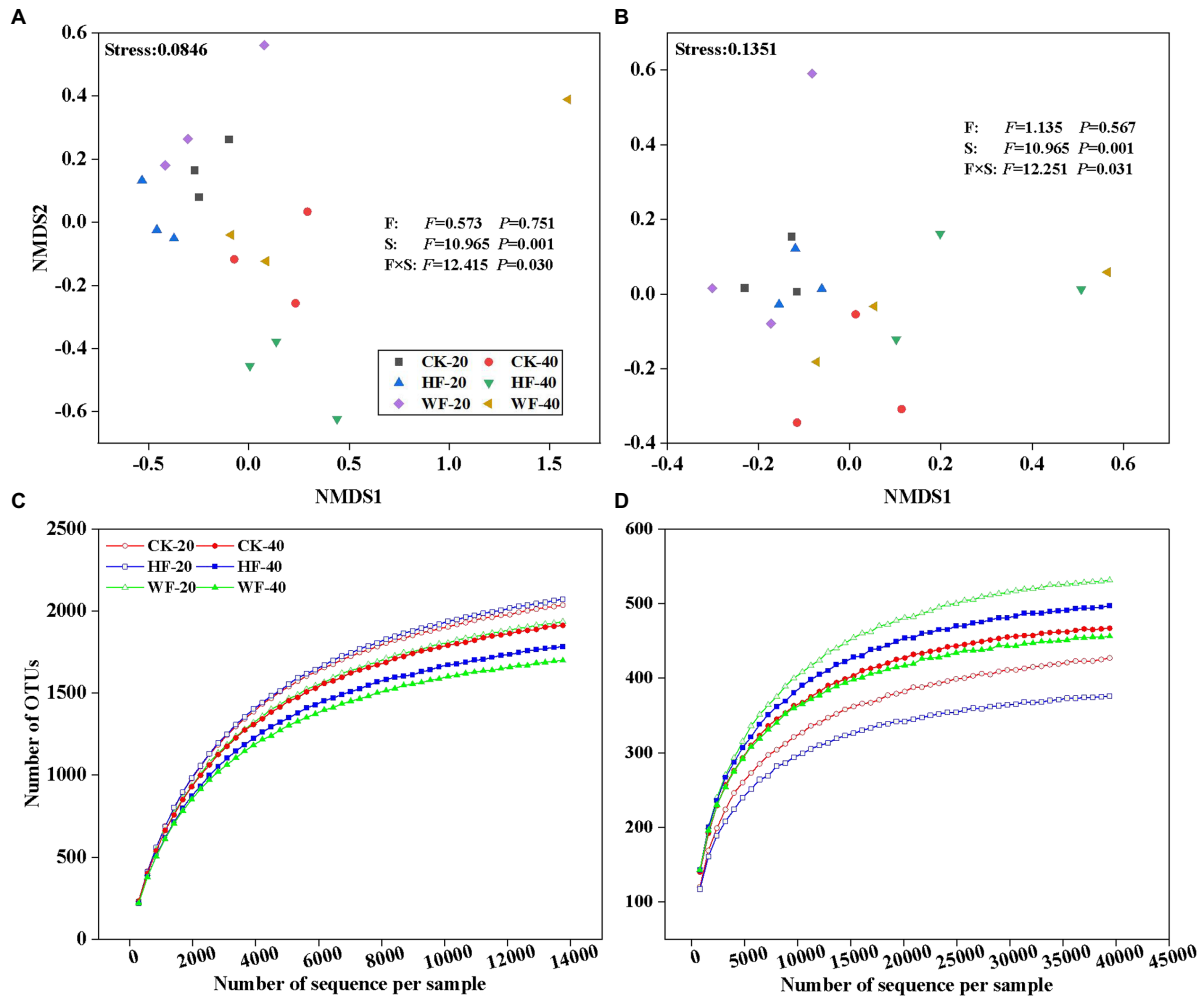
Non-metric multidimensional scaling (NMDS) ordination plots of soil bacteria **(A)** and fungi **(B)** compositional variation. Rarefaction curves of all samples are generated for bacteria **(C)** and fungi **(D)** OTUs, which contain unique sequences and are defined at 99% sequence similarities. These plots show the effects of fertilization and soil layers on microbial compositional variation. The compositional variation is represented with Bray-Curtis distance based on the abundance of OTUs. F, fertilization, including CK, HF, and WF; CK, no fertilization; HF, hole fertilization; WF, integration of water and fertilizer; S, soil layer, including S20 and S40; S20, surface soil (0–20  cm); S40, subsurface soil (20–40 cm); F × S, the interaction of fertilization and soil layer; CK-20, no fertilization with 0–20 cm soil layer; HF-20, hole fertilization with 0–20  cm soil layer; WF-20, integration of water and fertilizer with 0–20  cm soil layer; CK-40, no fertilization with 20–40 cm soil layer; HF-40, hole fertilization with 20–40 cm soil layer; WF-40, integration of water and fertilizer with 20–40 cm soil layer. *F-and p*-values under different treatments are from the results of the Kruskal-Wallis test.

The bacterial composition was dominated by Proteobacteria (34.2%), Acidobacteria (19.5%), and Actinobacteria (13.7%) across all fertilization regimes, but other taxa were present in lower abundance (Supplementary Figure S2 A). Our results revealed a strong shift in the abundance of four bacterial phyla in all treatments (Figure 2). For example, the abundance of GAL15, Nitrospirae, and WS3 was higher in the subsoil than in the topsoil, while Bacteroidetes exhibited the opposite trend. Our results further showed that there were 17 fungi phyla found in the *C. bungei* soil. The dominant phyla of fungi were Ascomycota, Basidiomycota, and Mortierellomycota, accounting for about 93.2% of all fungi abundance (Supplementary Figure S2 B). Rozellomycota was detected with low abundance, and the unclassified and rare phyla accounted for about 6.0% of all samples. Fertilization increased the abundance of Rozellomycota and Basidiomycota, but decreased the abundance of Ascomycota and Mortierellomycota in the subsoil. In addition, fertilization increased the abundance of Ascomycota and reduced the abundance of Basidiomycota in the topsoil (Figure 3). Bray–Curtis-based NMDS revealed that soils from the two depths harbored distinct bacterial and fungal communities, but the microbial composition did not undergo a significant shift upon fertilization addition. The interaction of fertilization and soil layer significantly altered the composition of soil bacterial and fungal communities (Figures 4A,B).

For bacterial species, *Silanimonas lenta* was significantly enriched with the highest abundance among all treatments. The abundances of *S. lenta* and *Paracoccus marcusii* were significantly different in the subsoil, while the abundances of *Duganella nigrescens*, *Nitrosovibrio tenuis*, *Methylobacterium hispanicum*, *Methylobacterium adhaesivum*, and *Blastococcus aggregatus* were significantly different in the topsoil without fertilization (Supplementary Figure S3 A). For fungal species, the abundances of *Thermomyces lanuginosus*, *Cladorrhinum* sp., and *Cladophialophora sp.* were significantly different in the topsoil under water and fertilizer integration conditions. The abundances of *Didymella macrostoma* and *Fusidium sp.* were significantly different in the subsoil without fertilization (Supplementary Figure S3 A).

### Bacterial and fungal diversity in response to fertilization

**Figure 5:**
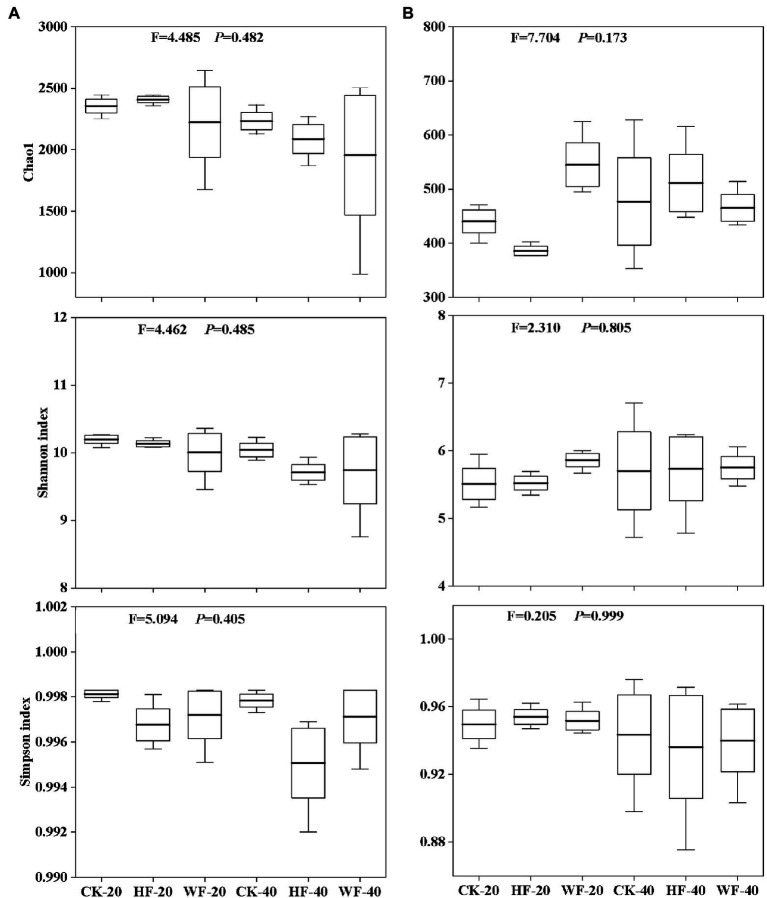
Diversity index of soil bacteria **(A)** and fungi **(B)**. CK-20, no fertilization with 0–20 cm soil layer; HF-20, hole fertilization with 0–20 cm soil layer; WF-20, integration of water and fertilizer with 0–20 cm soil layer; CK-40, no fertilization with 20–40 cm soil layer; HF-40, hole fertilization with 20–40 cm soil layer; WF-40, integration of water and fertilizer with 20–40  cm soil layer. The thick line in the boxplot represents the mean. F- and *p*-values are both from the results of the Kruskal-Wallis test.

Fertilization had a negative impact on the number of bacterial OTUs in the subsoil, but had a less pronounced effect on that in the topsoil (Figure 4C). The number of fungal OTUs was not affected by fertilization in the subsoil, but the integration of water and fertilizer (WF) significantly increased the number of OTUs compared with no fertilization (CK) and hole fertilization (HF) in the topsoil (Figure 4D). The results found that fertilization reduces the bacteria’s Chao1, Shannon, and Simpson indices in both soil layers compared with no fertilization (Figure 5A). In addition, the bacterial diversity was also affected by soil depth. For example, the Chao1 and Shannon indices of bacteria in the topsoil were higher than those in the subsoil. Fertilization could increase the fungal diversity slowly (e.g., Chao1 and Shannon indices) in the topsoil, but had no effects on that in the subsoil (Figure 5B).

### Functional groups of bacteria and fungi in response to fertilization

The PICRUSt2 analysis was used to generate hypotheses about bacterial pathways that might be altered by the treatments in this study. Our initial findings believed that all samples involved a total of six types of metabolic pathways: cellular processes, environmental information processing, genetic information processing, human diseases, metabolism, and organismal systems (Supplementary Figure S4). Among them, metabolism was the main function with an abundance of 73.4%, while the proportion of the other five functional types was only 2.3–10.3% (Supplementary Table S2). The analysis of the predicted functions of bacterial communities revealed that the abundance of the six primary functional groups did not differ significantly among all treatments. Further analysis of the predicted genes in the secondary functional taxonomy showed that a total of 47 sub-functions were identified, including amino acid metabolism (11.4%), metabolism of cofactors and vitamins (10.0%), carbohydrate metabolism (9.3%), metabolism of other amino acids (6.7%), and so on (Supplementary Table S3). None of these sub-functional groups were significantly affected by all treatments. The lack of significance for the PICRUSt2 results might be due to its development for human microbiome analysis and known issues when applying it to environmental samples.

The fungal functions of all treatments were compared using the FUNGuild database. The trophic mode of fungi was dominated by symbiotroph, exceeding 85.0% in abundance, while the abundance of other types did not exceed 6.0%, such as saprotroph (3.6%) and pathotroph (0.2%; Supplementary Figure S5). Fertilization decreased the abundance of symbiotroph but increased that of saprotroph in both soil layers (Supplementary Table S4).

### Correlation between bacterial and fungal abundance and soil properties

**Figure 6:**
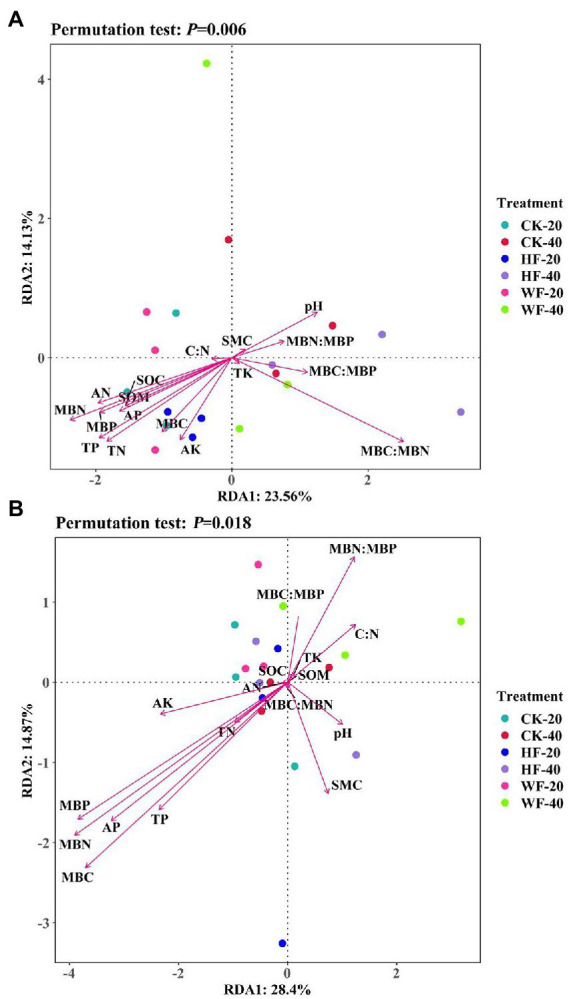
Redundancy analysis (RDA) of soil properties and relative abundance of bacteria **(A)** and fungi **(B)** from different treatments. SMC, soil moisture content; SOM, soil organic matter; SOC, soil organic carbon; TN, total nitrogen; TP, total phosphorus; TK, total potassium; AN, available nitrogen; AP, available phosphorus; AK, available potassium; C:N ratio, the ratio between the SOC and TN; MBC, microbial biomass carbon; MBN, microbial biomass nitrogen; MBP, microbial biomass phosphorus; CK-20, no fertilization with 0–20 cm soil layer; HF-20, hole fertilization with 0–20 cm soil layer; WF-20, integration of water and fertilizer with 0–20 cm soil layer; CK-40, no fertilization with 20–40 cm soil layer; HF-40, hole fertilization with 20–40 cm soil layer; WF-40, integration of water and fertilizer with 20–40  cm soil layer.

The relationships between microbial abundance and soil properties were shown in the RDA (Figure 6; Supplementary Table S5). The first ordination (RDA1 axis) of bacteria was mainly correlated with pH, SOM, SOC, TN, TP, AN, AP, AK, MBC, MBN, MBP, and MBC:MBN ratio, and explained 23.56% of the total variances. The abundance of fungi was only closely related to AP, MBC, MBN, and MBP. Correlations between soil properties showed that pH was significantly positively correlated with catalase but negatively correlated with TN, AN, AP, AK, MBP, and sucrase. Furthermore, SOC, TN, and TP were found to respond positively to AN, MBC, MBN, and sucrase (Supplementary Figure S6).

### Network analysis of soil bacterial and fungal community

**Figure 7:**
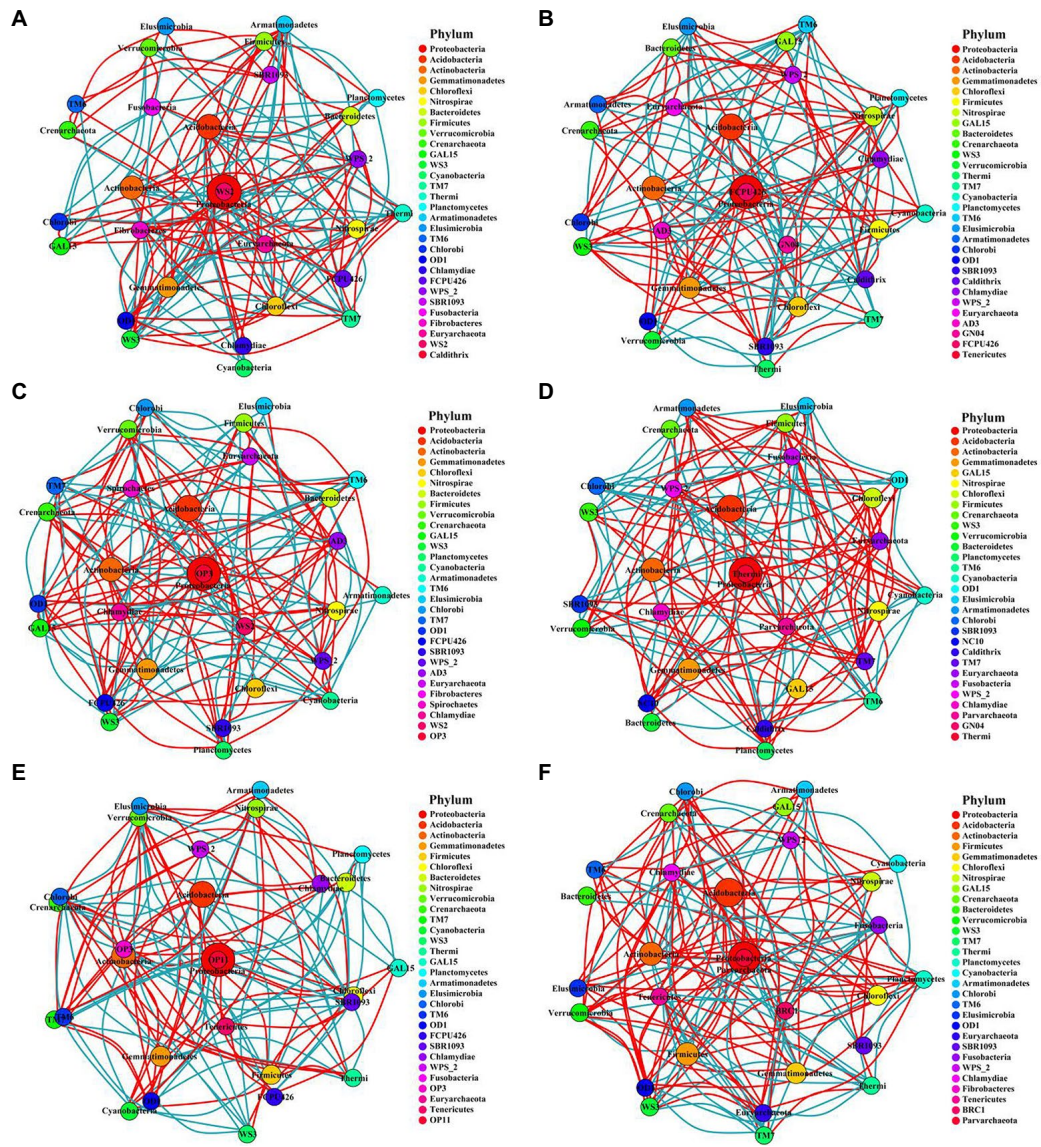
Networks of bacterial communities in different fertilization treatments at the phylum level. The circle represents phyla, and the circle size represents its relative abundance. The circle colors represent different phyla, and the line between the circles represents a significant correlation between the two phyla (*p* < 0.05; Spearman’s correlation). The red line represents a positive correlation, and the blue line represents a negative correlation. The thicker the line, the greater the absolute value of the correlation coefficient. **(A)** CK-20, no fertilization with 0–20 cm soil layer; **(B)** CK-40, no fertilization with 20–40 cm soil layer; **(C)** HF-20, hole fertilization with 0–20 cm soil layer; **(D)** HF-40, hole fertilization with 20–40 cm soil layer; **(E)** WF-20, integration of water and fertilizer with 0–20 cm soil layer; **(F)** WF-40, integration of water and fertilizer with 20–40  cm soil layer.

**Figure 8:**
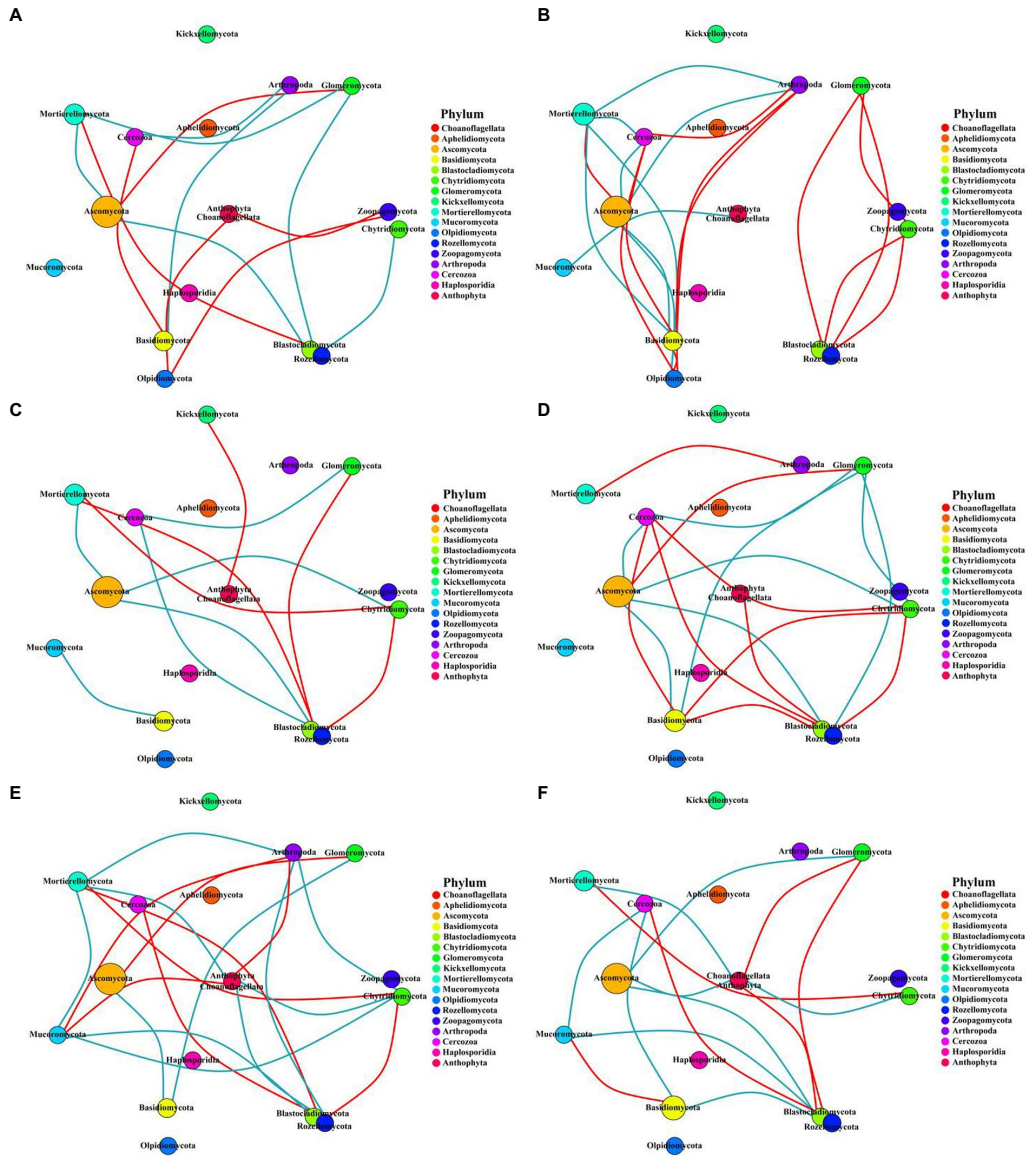
Networks of fungi communities in different fertilization treatments at the phylum level. The circle represents phyla, and the circle size represents its relative abundance. The circle colors represent different phyla, and the line between the circles represents a significant correlation between the two phyla (*p* < 0.05; Spearman’s correlation). The red line represents a positive correlation, and the blue line represents a negative correlation. The thicker the line, the greater the absolute value of the correlation coefficient. **(A)** CK-20, no fertilization with 0–20 cm soil layer; **(B)** CK-40, no fertilization with 20–40 cm soil layer; **(C)** HF-20, hole fertilization with 0–20 cm soil layer; **(D)** HF-40, hole fertilization with 20–40  cm soil layer; **(E)** WF-20, integration of water and fertilizer with 0–20 cm soil layer; **(F)** WF-40, integration of water and fertilizer with 20–40 cm soil layer.

This study constructed the networks of the microbial communities *via* taking advantage of correlation relationships between the abundance of microbe phylum to study the interaction modes in biological systems. Although the number of nodes for bacteria or fungi were consistent across all treatments, the number of links varied widely (Supplementary Table S6). The number of bacterial or fungal links was higher in the two fertilization regimes than in the control. However, the number of links between microbes in the subsoil was lower than in the topsoil. In addition, the number of nodes and links of bacteria was much higher than that of fungi, indicating that the connectivity of bacteria was more complicated while the connectivity of fungi was much simpler in all the fertilization circumstances (Figures 7, 8).

### Pathways determining the diversity and composition of soil bacterial and fungal community

**Figure 9:**
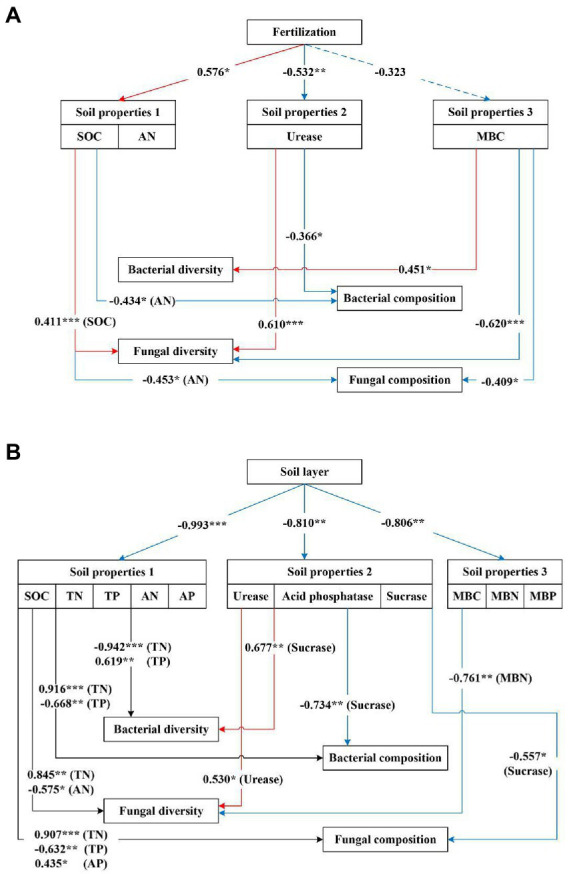
**(A)** Structural equation models (SEM) described the effects of fertilization and soil properties on the diversity and composition of soil bacterial and fungal communities. For the model, *p* = 0.687, RMSEA<0.05, CFI = 0.979; GFI = 0.990. **(B)** Structural equation models (SEM) described the effects of the soil layer and soil properties on the diversity and composition of soil bacterial and fungal communities. For the model, *p* = 0.586, RMSEA<0.05, CFI = 0.964; GFI = 0.987. SOC, soil organic carbon; TN, total nitrogen; TP, total phosphorus; AN, available nitrogen; AP, available phosphorus; MBC, microbial biomass carbon; MBN, microbial biomass nitrogen; MBP, microbial biomass phosphorus. Path coefficients only indicated significant standardized prediction coefficients. Red pathways indicated significant positive paths, and blue pathways indicated significant negative paths. Solid lines indicated significant paths (^*^p < 0.05; ^**^p < 0.01; ^***^p < 0.001), and dashed lines indicated not significant paths (*p* > 0.05).

The SEM model indicated that fertilization indirectly affected the diversity and composition of soil bacterial and fungal communities by directly affecting the soil properties (Figure 9A; Supplementary Table S7). Fertilization indirectly affected the bacterial composition through its negative effects on urease and positive effects on AN. It also had an indirect positive effect on the diversity of the fungal community through the effects of fertilization on SOC, and fungal community composition was negatively affected by AN and MBC. The soil layer had more complex effects on bacterial and fungal diversity and composition than fertilization (Figure 9B; Supplementary Table S8). The results showed that the soil layer exerted indirect effects on bacterial diversity and composition by primarily affecting the nutrient contents (including TN and TP). Fungal diversity was positively affected by TN but negatively affected by AN and MBN. In addition, the soil layer indirectly altered the fungal composition *via* its effect on TN and AP.

## Discussion

### Effects of fertilization on the diversity and composition of soil microbial community

Based on the 4-year fertilization experiments in the *C. bungei* plantation, we observed that the diversity of the bacterial community, such as Chao1, Shannon, and Simpson indices, had a decreasing trend in the topsoil when undergoing fertilization compared with no fertilization (Figure 5A). The findings of this study were confirmed by previous studies. These results believed that long-term fertilization reduced bacterial species and richness *via* aggravating soil acidification (Curtin et al., 1998; Yao et al., 2014; Chen et al., 2015). We also found that bacterial diversity gradually decreased with the deepening of the soil layer, which was consistent with previous studies that showed lower bacterial diversity in the subsoil of the Eucalyptus plantation than in the topsoil (Lin et al., 2022). The reason for this result might be due to less accumulation of elemental contents (e.g., N and P) in the deeper soil (Wang et al., 2017). We further noticed that there was little differentiation in the composition of bacterial or fungal communities among different fertilization regimes (Figures 4A,B). In contrast, there is a greater difference in the structures of bacterial or fungal communities in both soil layers. The reason might be that the topsoil was enriched with a large amount of litter, sufficient nutrients, and high root activity, which was conducive to improving biomass and activity of soil microbes, while the decreased metabolic activity of soil microbes was caused by the deterioration of the subsoil habitat conditions (Wu et al., 2013).

In our experiments, two types of fertilizations (i.e., the integration of water and fertilizer and hole fertilization) had contrasting effects on the soil parameters and structure of the soil microbial community. For example, most soil indicators undergoing the integrated water and fertilizer measures, such as total N, total P, and soil enzyme activities, showed a slight descending trend compared with hole fertilization (Table 1). We further observed that the bacterial diversity (e.g., Chao1 and Shannon indices) in the topsoil undergoing the coupled water and fertilizer conditions was lower than the hole application (Figure 5A). These results might be due to the following reasons. On the one hand, the uptake efficiency of water-soluble fertilizers was higher than the solid fertilizers (i.e., hole fertilization) in tree populations (Feng et al., 2019; Fan et al., 2020). On the other hand, the vigorous growth of young *C. bungei* clones undergoing drip fertigation (i.e., the integration of water and fertilizer) depleted a large amount of nutrition from the soil, resulting in a decrease in the size of the soil nutrient pool.

The SEM results indicated that soil properties could explain the effects of fertilization or soil layer on microbial communities. Our results suggested that bacterial and fungal compositions undergoing fertilization were mainly related to available N, while fungal diversity was mainly affected by the urease and soil organic carbon (Figure 9A). However, Ling et al. (2017) found that nutrition additions affected the bacterial community primarily by changing the pH and improving P availability. The results of this study contradicted our experiment due to the difference in the amount of fertilizer, the type of fertilizer, and the time of application. We further observed that bacterial diversity and composition were closely related to total N and total P, while fungal diversity and composition were regulated by N and P contents at different soil depths (Figure 9B). Thus, the mechanisms driving the fungal and bacterial responses to fertilization and soil layer might be different, and as a result, the soil nutrients that affected fungi and bacteria were also different.

### Effects of fertilization on the function of soil microbial community

Our experiment observed that all bacterial taxa involved six types of metabolic pathways and had 47 sub-functions, showing rich functional diversity (Supplementary Figure S4). However, we found no fertilizer effect on the primary and secondary functional profiles. This supported functional redundancy as a mechanism for the stabilization of functions during changes in microbial composition (Langenheder et al., 2010; Frossard et al., 2012). Functional redundancy referred to the hypothesis that each member of the community had the same functional capability, such that if the composition of the community changed, the metabolic output did not. We further discovered that the major functional genes were all metabolism-related functions. This result was in line with the reports of Lin et al. (2022), who found that metabolism played an extremely pivotal role in the soil of Eucalyptus plantations. Furthermore, no significant differences were found in bacterial functional groups between the topsoil and subsoil (Supplementary Tables S2, S3), indicating that soil depth might not be the main factor affecting bacterial function. The reason for this result needed to be further explored.

We also noted that symbiotroph and saprotroph abundances predominated across all fertilization regimes (Supplementary Figure S5). However, fertilization decreased the abundance of symbiotic fungi but increased the abundance of saprophytic fungi. Our result was in contrast to the observations of Chen et al. (2020). They found that short-term fertilization (6 months) significantly increased the enrichment of symbiotic fungi in the Eucalyptus soil, while decreasing the abundance of saprophytic fungi, animal, and plant pathogens. The reason for this difference might be related to the time of fertilizer accumulation and the biological differences between evergreen and deciduous species. Ectomycorrhizal fungi were regarded as key elements of forest nutrient cycles and strong drivers of forest ecosystem processes (Anderson and Cairney, 2010). For example, Basidiomycetes was involved in complex C cycling in different forest types by biosynthesizing several enzymes to decompose wood (primarily wood with high levels of complex C compounds; Chen et al., 2014). However, we found that fertilization (especially the integration of water and fertilizer) reduced the abundance of Basidiomycetes in the soil. The results of Li et al. (2022b) suggested that ectomycorrhizal fungi and saprophytes were mutually antagonistic, such as the presence of ectomycorrhizal fungi might decrease saprotrophic fungi abundance *via* suppressing the activities of saprotrophic fungi in regulating litter decomposition. Furthermore, Klaus et al. (2018) also found that free-living saprophytes might become dominant and potentially alter soil C and N cycles with the loss of ectomycorrhizal fungi. Bödeker et al. (2016) reported that saprotrophic fungi often dominated in the humus layer and overlapped fundamental niches with ectomycorrhizal fungi. These results confirmed our findings that fertilization increased the abundance of saprophytic fungi (e.g., Ascomycota) and decreased the abundance of ectophytic fungi (e.g., Basidiomycetes). We further found that symbiotic fungi were mainly enriched in the topsoil, while saprophytic fungi were mostly distributed in the subsoil. However, our study was not yet able to explain these effects of soil layer on fungal function in detail. Therefore, it might be quite necessary to investigate the response mechanisms of soil fungal communities to soil profiles to increase ecosystem balance and stability in future studies (Karst et al., 2015; Klaus et al., 2018).

The experiment constructed the networks of the microbial community to study the interaction modes in biological systems (Zhou et al., 2011; Chen et al., 2019). Ascomycota of the fungi phylum played a keystone role in fungal networks in our findings (Figure 8), which was similar to a previous study (Barberán et al., 2012). While in all bacterial phyla, we found that the Acidobacteria, Proteobacteria, and Verrucomicrobia phylum played module-hub or connector roles in the networks (Figure 7). Fertilization (especially the integration of water and fertilizer) could enhance the association between bacterial or fungal taxa (Supplementary Table S6), which helped to maintain the stability of the ecosystem.

## Conclusion

Collectively, fertilization regimes and soil profiles had different effects on soil properties and microorganisms. The integration of water and fertilizer reduced nutrient availability, bacterial diversity, and abundance of symbiotic fungi but increased saprophytes taxa in the soil compared with hole fertilization. The soil nutrient contents, microbial diversity, and symbiotic fungal taxa decreased with the deepening of the soil layer. Soil profiles altered the bacterial and fungal community structure, but fertilization did not. The main factor affecting bacterial and fungal composition was available N, and the main factors affecting fungal diversity were urease activity and soil organic carbon undergoing fertilization. The soil layer mainly affected the community structure of bacteria and fungi by changing the contents of N and P in the soil. Therefore, the appropriate regulation of soil properties would be an important approach for maintaining a high-level soil microbial community in young *C. bungei* stand.

## Data availability statement

The data presented in the study are deposited in the Sequence Read Archive (SRA) and assigned the following BioProject accession number: PRJNA847783 and PRJNA848013.

## Author contributions

ZG: manuscript writing. DL, DC, YL, QH, and NL: sample collection. QQ: data collection. JW and YS: data analysis. YG: mapping. JL: manuscript revision. WM and QH: manuscript check. All authors contributed to the article and approved the submitted version.

## Funding

This research was supported by grants from the National Key Research and Development Program of China (2017YFD0600604 and 2017YFD060060404).

## Conflict of interest

The authors declare that the research was conducted in the absence of any commercial or financial relationships that could be construed as a potential conflict of interest.

## Publisher’s note

All claims expressed in this article are solely those of the authors and do not necessarily represent those of their affiliated organizations, or those of the publisher, the editors and the reviewers. Any product that may be evaluated in this article, or claim that may be made by its manufacturer, is not guaranteed or endorsed by the publisher.

## Footnotes

^1^ https://docs.qiime2.org/2019.1/

^2^ https://www.cloudtutu.com

## Supplementary material

The Supplementary material for this article can be found online at: https://www.frontiersin.org/articles/10.3389/fmicb. 2022.948875/full#supplementary-material
