# Supplementary material for: Soil microbial communities response to different fertilization regimes in young Catalpa bungei plantation
Source: Front Microbiol. 2022 Aug 8;13:948875. doi: 10.3389/fmicb.2022.948875 (PMC9473346; doi:10.3389/fmicb.2022.948875)
Supplement: Supplementary file 1 [file Data_Sheet_1.doc]

**Supplementary material**

Figure S1. Monthly precipitation and monthly average temperature from 2017 to 2021, measured in Zhangqiu District, China.

Figure S2. Relative abundance of bacteria (a) and fungi group (b) at phylum level under different fertilization treatments.

Note: The group accounting for ≥1% are shown while those <1% and unclassified group are integrated into ‘others’. CK-20, no fertilization with 0-20 cm soil layer; HF-20, hole fertilization with 0-20 cm soil layer; WF-20, integration of water and fertilizer with 0-20 cm soil layer; CK-40, no fertilization with 20-40 cm soil layer; HF-40, hole fertilization with 20-40 cm soil layer; WF-40, integration of water and fertilizer with 20-40 cm soil layer.


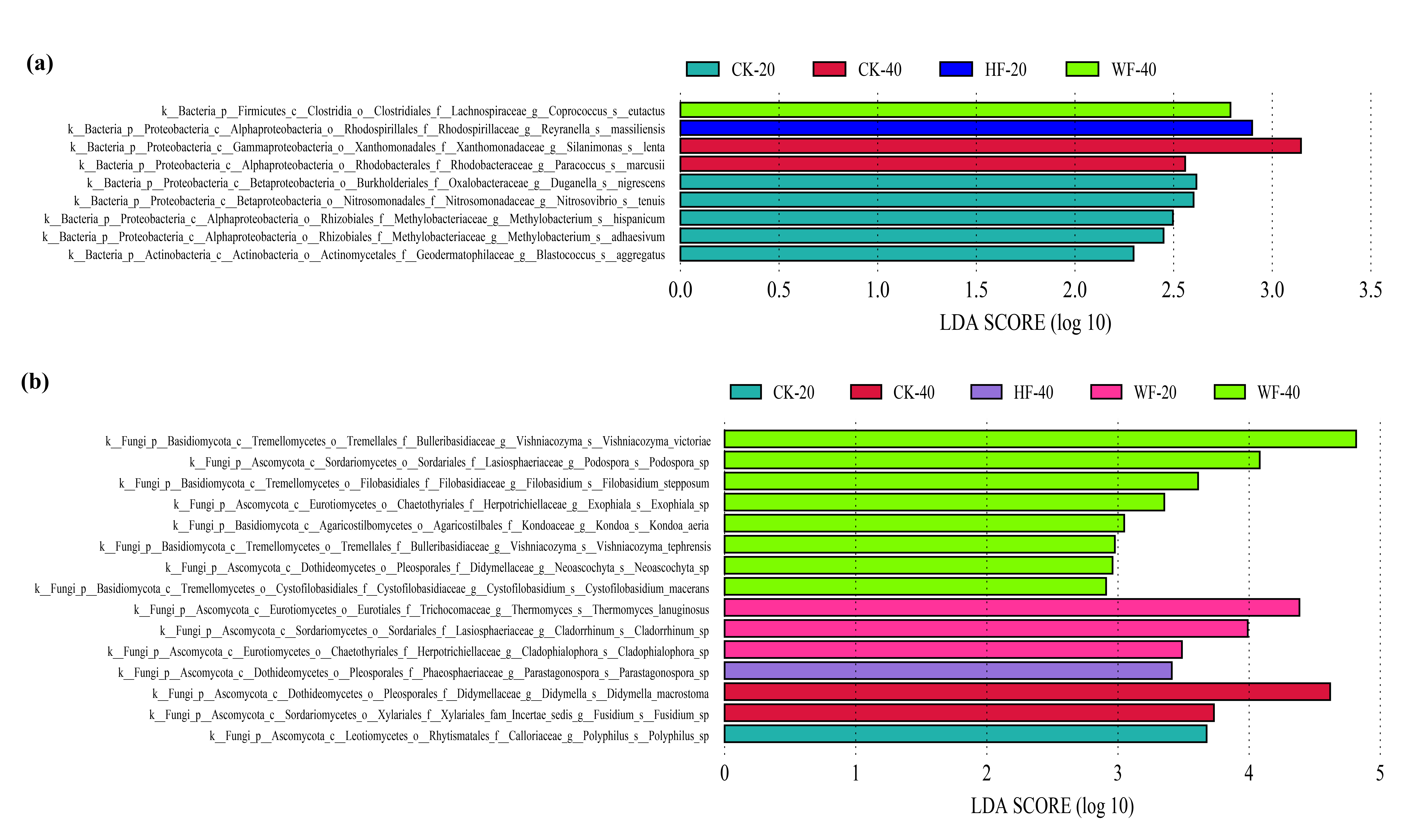


Figure S3 Linear discriminant analysis (LDA) of soil bacterial (a) and fungal communities (b).


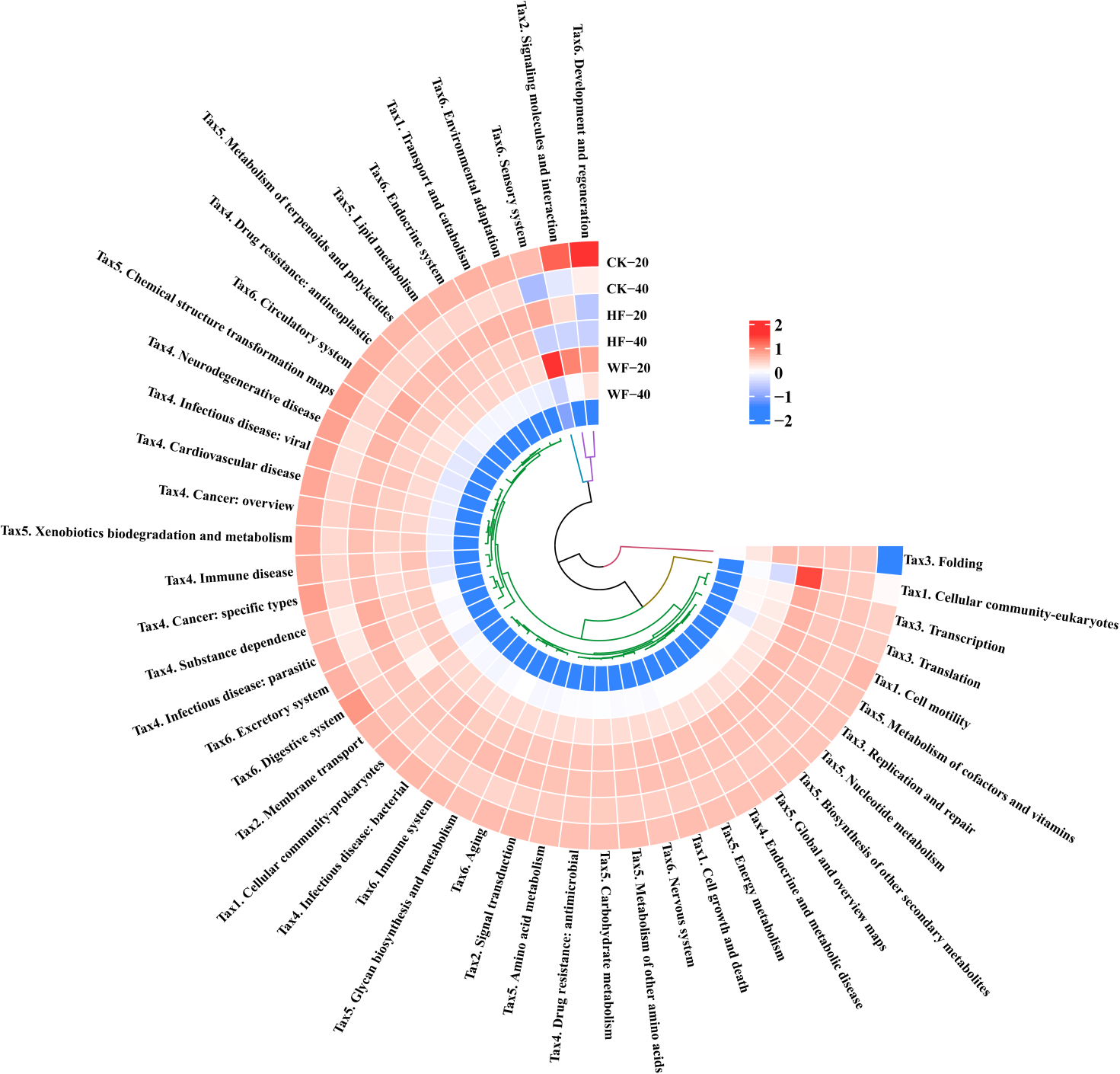


Figure S4. Heat map of PICRUSt2 based function predicted for bacterial taxonomy.

Note: Tax1: cellular processes; Tax2: environmental information processing; Tax3: genetic information processing; Tax4: human diseases; Tax5: metabolism; Tax6: organismal systems. CK-20, no fertilization with 0-20 cm soil layer; HF-20, hole fertilization with 0-20 cm soil layer; WF-20, integration of water and fertilizer with 0-20 cm soil layer; CK-40, no fertilization with 20-40 cm soil layer; HF-40, hole fertilization with 20-40 cm soil layer; WF-40, integration of water and fertilizer with 20-40 cm soil layer.

Figure S5. Compositions of fungal functional group (guild) inferred by FUNGuild under different fertilization.

Note: CK-20, no fertilization with 0-20 cm soil layer; HF-20, hole fertilization with 0-20 cm soil layer; WF-20, integration of water and fertilizer with 0-20 cm soil layer; CK-40, no fertilization with 20-40 cm soil layer; HF-40, hole fertilization with 20-40 cm soil layer; WF-40, integration of water and fertilizer with 20-40 cm soil layer. The break is based on the median value of relative abundance.

Figure S6. Spearman’s correlation analysis of soil properties among different treatments.

Note: SMC, soil moisture content; SOM, soil organic matter; SOC, soil organic carbon; TN, total nitrogen; TP, total phosphorus; TK, total potassium; AN, available nitrogen; AP, available phosphorus; AK, available potassium; C:N ratio, the ratio between the SOC and TN. MBC, microbial biomass carbon; MBN, microbial biomass nitrogen; MBP, microbial biomass phosphorus.
