# Supplementary material for: Soil microbial communities response to different fertilization regimes in young Catalpa bungei plantation
Source: Front Microbiol. 2022 Aug 8;13:948875. doi: 10.3389/fmicb.2022.948875 (PMC9473346; doi:10.3389/fmicb.2022.948875)
Supplement: Supplementary file 2 [file Data_Sheet_2.doc]

**Supplementary material**

Table S1. Growth of *[Catalpa](../../../../D:/Dict/8.9.6.0/resultui/html/index.html#/javascript:;) [bungei](../../../../D:/Dict/8.9.6.0/resultui/html/index.html#/javascript:;)* plantation with different fertilization, measured in October 2021

| Fertilization | Tree height (m) | |  | DBH (cm) | |  | Crown diameter (m) | |
| --- | --- | --- | --- | --- | --- | --- | --- | --- |
|  | Min.-Max. | Mean±SD |  | Min.-Max. | Mean±SD |  | Min.-Max. | Mean±SD |
| CK | 6.5-9.6 | 8.5±0.63 |  | 9.85-14.12 | 12.74±0.99 |  | 2.35-4.45 | 2.91±0.40 |
| HF | 8.1-9.7 | 8.9±0.41 |  | 12.41-14.89 | 13.62±0.66 |  | 2.65-4.40 | 3.22±0.37 |
| WF | 7.1-10.0 | 9.1±0.58 |  | 13.43-16.80 | 15.25±0.82 |  | 2.80-4.55 | 3.50±0.34 |

Note: CK, no fertilization; HF, hole fertilization; WF, [integration](../../../../D:/Dict/8.9.6.0/resultui/html/index.html#/javascript:;) [of](../../../../D:/Dict/8.9.6.0/resultui/html/index.html#/javascript:;) [water](../../../../D:/Dict/8.9.6.0/resultui/html/index.html#/javascript:;) [and](../../../../D:/Dict/8.9.6.0/resultui/html/index.html#/javascript:;) [fertilizer](../../../../D:/Dict/8.9.6.0/resultui/html/index.html#/javascript:;); DBH, diameter at breast height; SD, [standard](../../../../D:/Dict/8.9.6.0/resultui/html/index.html#/javascript:;) [deviation](../../../../D:/Dict/8.9.6.0/resultui/html/index.html#/javascript:;).

Table S2. Functional analysis of soil bacteria among different fertilization.

| Bacterial function | CK | |  | HF | |  | WF | | Total reads | K-W test |
| --- | --- | --- | --- | --- | --- | --- | --- | --- | --- | --- |
|  | S20 | S40 |  | S20 | S40 |  | S20 | S40 |  |  |
| Cellular Processes | 105961±3329 | 100162±7939 |  | 103292±5599 | 104014±11441 |  | 93268±22658 | 78625±46777 | 1755969 | ns |
| Environmental Information Processing | 37158±527 | 35162±2239 |  | 35848±1601 | 35482±3239 |  | 33636±7921 | 28376±16382 | 616990 | ns |
| Genetic Information Processing | 155820±7226 | 156654±15150 |  | 157441±5289 | 162925±10801 |  | 140934±44313 | 130575±79838 | 2713053 | ns |
| Human Diseases | 78722±2157 | 72399±5169 |  | 75911±4751 | 74165±9732 |  | 70093±18266 | 58121±34443 | 1288234 | ns |
| Metabolism | 1150086±49937 | 1092953±93390 |  | 1133781±52256 | 1124495±112882 |  | 1041463±310344 | 900779±548147 | 19330679 | ns |
| Organismal Systems | 37928±1678 | 34772±3068 |  | 37376±1966 | 35656±4378 |  | 34246±10316 | 28436±17418 | 625245 | ns |

Note: Mean±SE were shown. CK, no fertilization; HF, hole fertilization; WF, integration of water and fertilizer. S, soil layer, including S20 and S40. S20, surface soil (0-20 cm); S40, subsurface soil (20-40 cm). ns, no significant (Kruskal-Wallis test).

Table S3. Sub-functional analysis of soil bacteria among different fertilization.

| Rank | Bacterial sub-function | CK | |  | HF | |  | WF | | Total reads | K-W test |
| --- | --- | --- | --- | --- | --- | --- | --- | --- | --- | --- | --- |
|  |  | S20 | S40 |  | S20 | S40 |  | S20 | S40 |  |  |
| 1 | Amino acid metabolism | 177970±7817 | 170798±14869 |  | 175873±7638 | 176166±16490 |  | 161707±48799 | 141288±85969 | 3011404 | ns |
| 2 | Metabolism of cofactors and vitamins | 154318±6007 | 151637±14146 |  | 154017±5498 | 157145±12002 |  | 138852±41720 | 125004±77120 | 2642922 | ns |
| 3 | Carbohydrate metabolism | 144279±5272 | 139001±10537 |  | 142189±5614 | 142074±12789 |  | 131982±38914 | 115655±69030 | 2445539 | ns |
| 4 | Metabolism of other amino acids | 104719±4206 | 99461±7982 |  | 103089±4724 | 101786±10805 |  | 94603±27818 | 82082±49886 | 1757218 | ns |
| 5 | Biosynthesis of other secondary metabolites | 93703±4519 | 91080±8431 |  | 93623±3035 | 92861±7637 |  | 85426±26976 | 76304±46841 | 1598991 | ns |
| 6 | Global and overview maps | 91431±4016 | 89211±7856 |  | 91017±3679 | 92213±7717 |  | 83096±25146 | 74035±44997 | 1563009 | ns |
| 7 | Lipid metabolism | 93327±4858 | 84515±8085 |  | 91620±6095 | 87677±13160 |  | 84737±24872 | 69311±42487 | 1533563 | ns |
| 8 | Xenobiotics biodegradation and metabolism | 75594±3502 | 65314±6363 |  | 70973±5305 | 66925±11419 |  | 68051±17426 | 51814±30890 | 1196011 | ns |
| 9 | Replication and repair | 66524±2959 | 66043±5786 |  | 66613±2362 | 67904±4723 |  | 60218±18540 | 54668±33051 | 1145911 | ns |
| 10 | Energy metabolism | 65258±2714 | 63759±5355 |  | 64393±2326 | 65371±4613 |  | 59041±17562 | 52665±31797 | 1111459 | ns |

Note: Mean±SE were shown. The number of total reads ranked in the top ten in the table. CK, no fertilization; HF, hole fertilization; WF, integration of water and fertilizer. S, soil layer, including S20 and S40. S20, surface soil (0-20 cm); S40, subsurface soil (20-40 cm). ns, no significant (Kruskal-Wallis test).

Table S4. Functional analysis of soil fungi among different fertilization.

| Fugal function | CK | |  | HF | |  | WF | | K-W test |
| --- | --- | --- | --- | --- | --- | --- | --- | --- | --- |
|  | S20 | S40 |  | S20 | S40 |  | S20 | S40 |  |
| Symbiotroph | 334.75±63.46 | 304.54±63.02 |  | 339.91±63.72 | 283.52±64.13 |  | 308.88±55.25 | 298.09±60.24 | ns |
| Saprotroph | 3.35±0.33b | 4.12±0.36ab |  | 3.41±0.33b | 4.21±0.37ab |  | 4.65±0.38a | 5.28±0.42a | ** |
| Pathotroph | 0.50±0.09b | 0.57±0.09b |  | 0.46±0.09b | 0.99±0.12a |  | 0.97±0.11a | 0.71±0.10ab | *** |

Note: Mean±SE were shown. The number of OTUs in all treatments in the table. CK, no fertilization; HF, hole fertilization; WF, integration of water and fertilizer. S, soil layer, including S20 and S40. S20, surface soil (0-20 cm); S40, subsurface soil (20-40 cm). ns, no significant. **, *P* <0.01; ***, *P* <0.001. Different lowercase letters in the same row indicated significant differences (*P* <0.05; Kruskal-Wallis test).

Table S5. The results of envfit function of R packages indicated correlations of soil parameters with overall microbial communities among all treatments.

| Soil properties | Bacteria | |  | Fungi | |
| --- | --- | --- | --- | --- | --- |
|  | R^2^ | *P* value |  | R^2^ | *P* value |
| SMC | 0.057 | 0.648 |  | 0.127 | 0.442 |
| pH | 0.345 | **0.043** |  | 0.107 | 0.575 |
| SOM | 0.419 | **0.015** |  | 0.061 | 0.997 |
| SOC | 0.419 | **0.015** |  | 0.061 | 0.997 |
| TN | 0.537 | **0.003** |  | 0.101 | 0.597 |
| TP | 0.554 | **0.002** |  | 0.182 | 0.224 |
| TK | 0.035 | 0.757 |  | 0.070 | 0.896 |
| AN | 0.506 | **0.005** |  | 0.064 | 0.959 |
| AP | 0.445 | **0.010** |  | 0.316 | **0.042** |
| AK | 0.343 | **0.040** |  | 0.152 | 0.326 |
| C:N ratio | 0.072 | 0.565 |  | 0.113 | 0.520 |
| MBC | 0.362 | **0.031** |  | 0.460 | **0.006** |
| MBN | 0.621 | **0.001** |  | 0.397 | **0.015** |
| MBP | 0.512 | **0.005** |  | 0.353 | **0.026** |
| MBC:MBN ratio | 0.684 | **0.000** |  | 0.064 | 0.960 |
| MBC:MBP ratio | 0.275 | 0.101 |  | 0.098 | 0.644 |
| MBN:MBP ratio | 0.196 | 0.196 |  | 0.139 | 0.383 |

Note: Bold numbers in the table indicate *P* <0.05. SMC, soil moisture content; SOM, soil organic matter; SOC, soil organic carbon; TN, total nitrogen; TP, total phosphorus; TK, total potassium; AN, available nitrogen; AP, available phosphorus; AK, available potassium; C:N ratio, the ratio between the SOC and TN. MBC, microbial biomass carbon; MBN, microbial biomass nitrogen; MBP, microbial biomass phosphorus.

Table S6. Total nodes and links of fungal and bacterial networks in different fertilization types

| Treatment | Bacteria | |  | Fungi | |
| --- | --- | --- | --- | --- | --- |
|  | Total nodes | Total links |  | Total nodes | Total links |
| CK-20 | 50 | 709 |  | 17 | 74 |
| CK-40 | 50 | 702 |  | 17 | 62 |
| HF-20 | 50 | 723 |  | 17 | 75 |
| HF-40 | 50 | 719 |  | 17 | 65 |
| WF-20 | 50 | 727 |  | 17 | 80 |
| WF-40 | 50 | 719 |  | 17 | 69 |

Note: “node” mean microbial phylum; “link” mean the relationship between microbial phylum. CK-20, no fertilization with 0-20 cm soil layer; CK-40, no fertilization with 20-40 cm soil layer; HF-20, hole fertilization with 0-20 cm soil layer; HF-40, hole fertilization with 20-40 cm soil layer; WF-20, integration of water and fertilizer with 0-20 cm soil layer; WF-40, integration of water and fertilizer with 20-40 cm soil layer.

Table S7. Correlation coefficients and *P* values of structural equation models (SEM) described the effects of fertilization and soil properties on diversity and composition of soil bacterial and fungal communities.

|  | Soil parameters | Correlation coefficient | *P* value |
| --- | --- | --- | --- |
| Fertilization ~ | Soil properties 1 | 0.576 | **0.014** |
|  | Soil properties 2 | -0.532 | **0.008** |
|  | Soil properties 3 | -0.323 | 0.148 |
|  |  |  |  |
| Bacterial diversity ~ | SOC | -0.208 | 0.298 |
|  | AN | 0.171 | 0.380 |
|  | Urease | 0.231 | 0.246 |
|  | MBC | 0.451 | **0.019** |
|  |  |  |  |
| Bacterial composition ~ | SOC | 0.239 | 0.198 |
|  | AN | -0.434 | **0.017** |
|  | Urease | -0.366 | **0.048** |
|  | MBC | -0.342 | 0.055 |
|  |  |  |  |
| Fungal diversity ~ | SOC | 0.411 | **0.001** |
|  | AN | -0.219 | 0.066 |
|  | Urease | 0.610 | **0.000** |
|  | MBC | -0.620 | **0.000** |
|  |  |  |  |
| Fungal composition ~ | SOC | 0.282 | 0.147 |
|  | AN | -0.453 | **0.017** |
|  | Urease | -0.106 | 0.586 |
|  | MBC | -0.409 | **0.028** |

Note: SOC, soil organic carbon; AN, available nitrogen; MBC, microbial biomass carbon. Bold numbers in the table indicate *P* <0.05.

Table S8. Correlation coefficients and *P* values of structural equation models (SEM) described the effects of soil layer and soil properties on diversity and composition of soil bacterial and fungal communities.

|  | Soil parameters | Correlation coefficient | *P* value |
| --- | --- | --- | --- |
| Fertilization ~ | Soil properties 1 | -0.993 | **0.000** |
|  | Soil properties 2 | -0.810 | **0.002** |
|  | Soil properties 3 | -0.806 | **0.002** |
|  |  |  |  |
| Bacterial diversity ~ | SOC | -0.193 | 0.259 |
|  | TN | -0.942 | **0.001** |
|  | TP | 0.619 | **0.009** |
|  | AN | 0.205 | 0.236 |
|  | AP | -0.284 | 0.126 |
|  | Urease | 0.261 | 0.151 |
|  | Acid phosphatase | 0.172 | 0.305 |
|  | Sucrase | 0.677 | **0.006** |
|  | MBC | -0.325 | 0.091 |
|  | MBN | 0.139 | 0.396 |
|  | MBP | 0.055 | 0.768 |
|  |  |  |  |
| Bacterial composition ~ | SOC | 0.200 | 0.245 |
|  | TN | 0.916 | **0.001** |
|  | TP | -0.668 | **0.006** |
|  | AN | -0.171 | 0.308 |
|  | AP | 0.098 | 0.547 |
|  | Urease | -0.282 | 0.128 |
|  | Acid phosphatase | -0.026 | 0.965 |
|  | Sucrase | -0.734 | **0.004** |
|  | MBC | 0.277 | 0.134 |
|  | MBN | -0.008 | 1.112 |
|  | MBP | -0.057 | 0.756 |
|  |  |  |  |
| Fungal diversity ~ | SOC | 0.366 | 0.066 |
|  | TN | 0.845 | **0.002** |
|  | TP | 0.125 | 0.442 |
|  | AN | -0.575 | **0.013** |
|  | AP | -0.008 | 0.999 |
|  | Urease | 0.530 | **0.018** |
|  | Acid phosphatase | -0.327 | 0.090 |
|  | Sucrase | -0.193 | 0.259 |
|  | MBC | -0.024 | 0.980 |
|  | MBN | -0.761 | **0.003** |
|  | MBP | 0.070 | 0.682 |
|  |  |  |  |
| Fungal composition ~ | SOC | 0.231 | 0.192 |
|  | TN | 0.907 | **0.001** |
|  | TP | -0.632 | **0.008** |
|  | AN | -0.280 | 0.130 |
|  | AP | 0.435 | **0.038** |
|  | Urease | -0.150 | 0.363 |
|  | Acid phosphatase | -0.088 | 0.592 |
|  | Sucrase | -0.557 | **0.015** |
|  | MBC | 0.328 | 0.089 |
|  | MBN | -0.214 | 0.219 |
|  | MBP | -0.365 | 0.067 |

Note: SOC, soil organic carbon; TN, total nitrogen; TP, total phosphorus; AN, available nitrogen; AP, available phosphorus; MBC, microbial biomass carbon; MBN, microbial biomass nitrogen; MBP, microbial biomass phosphorus. Bold numbers in the table indicate *P* <0.05.
